# Salvage of Upper Limb following a Severe Crushing Trauma: Immediate Reconstruction with a Free Flap and Subsequent Hyperbaric Oxygen Therapy

**DOI:** 10.1155/2009/568142

**Published:** 2009-05-27

**Authors:** M. P. Serra, P. Longhi

**Affiliations:** ^1^Department of Plastic and Reconstructive Surgery, Ospedali Riuniti di Bergamo, Largo Barozzi no. 1, 24122 Bergamo, Italy; ^2^University Hospital North Durham, Durham DH1 5TW, UK

## Abstract

A microsurgical latissimus dorsi flap was performed for resurfacing a large soft tissue defect of the forearm with exposure of the vital structures and contaminated wound. Early coverage of a defect is a generally accepted concept to achieve a better functional result. The authors present a case report where a free latissimus dorsi flap with subsequent hyperbaric oxygen therapy allowed a successful single stage reconstruction of this complex severely contaminated defect.

## 1. Introduction

Management of skin loss that occurs after severe trauma of the upper extremity continues to challenge reconstructive surgeons. 

Local flaps remain usually inadequate since donor sites in the upper limb are restricted and such defects are fairly large in size. 

When a significant area of soft tissue coverage is required, free flaps are the preferred choice, especially if infection is present. 

Muscle flaps [1, 2] are usually selected for their capacity to resist infection and provide a blood supply to the surrounding tissues. 

Among these, the latissimus dorsi (LD) is the best choice for the coverage of wide defects, due to its reliable anatomy, large surface area, and a long pedicle. 

The authors present a case report where a large soft tissue defect of the forearm with exposure of the vital structures was reconstructed by a microsurgical latissimus dorsi flap, followed by hyperbaric oxygen therapy. 

The combination of free flap with hyperbaric therapy allowed a successful treatment of a complex severely contaminated wound.

## 2. Case Report

**Figure 1:**
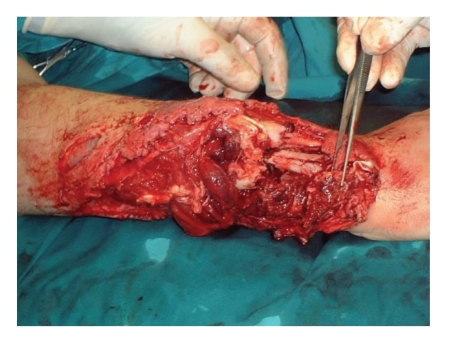
Preoperative crush injury right forearm.

A 17-year-old boy sustained a crush injury, in a contaminated environment, with extensive loss of dorsal and volar soft tissue involving 3/4 of his right forearm, complex displaced fractures of the radius and ulna, injury of the flexor tendons, median nerve, radial artery and laceration of the extensor tendons (Figure 1).

**Figure 2:**
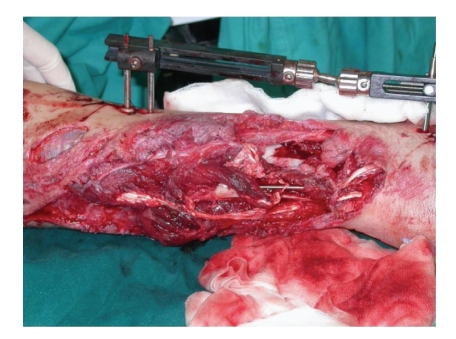
Intraoperative external and intramedullary fixations.

A combined procedure with the orthopaedic surgeon was therefore carried out as a matter of urgency. The orthopaedic timing consisted of a debridement of the wound and reduction of the fractures with external and intramedullary fixation (Figure 2), followed by a further debridement and washout of the wound with a pulsed lavage system, performed by the authors. The flexor and residual extensor tendons were repaired using, respectively, 3/0 and 4/0 nylon and a neurorraphy of the median nerve was carried out with 8/0 nylon under microscope magnification. 

The radial artery was injured at 2/3 of the forearm and its stump was tied at A&E. 

Following a removal of 1 cm thrombus, the proximal stump was trimmed for 4 cm, in order to be away from the zone of injury, but the radial artery had initially a low flow, due to the spasm of the vessel. One mL of papaverine was injected into the vessel and a few warm dumped swabs were wrapped around the forearm. 

Fifteen minutes later the situation improved and a good pulsation and outflow allowed us to consider this recipient vessel reliable for a free flap. The cephalic vein was then dissected and prepared. 

The choice of the flaps was restricted by the necessity to replace a large amount of skin and an LD flap was selected for its well known features and advantages to comply with an extensive soft tissue loss and infection.

An unilateral LD flap was harvested as the patient was left dominant hand. 

**Figure 3:**
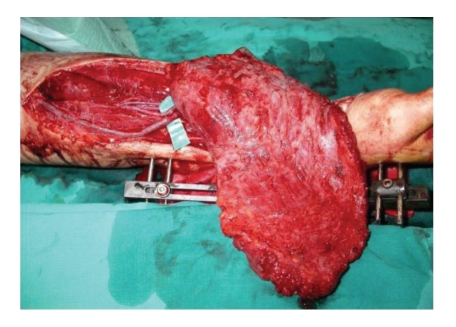
LD flap with anastomosis of the vessels.

The anastomosis was carried out between the radial artery and the toracodorsal artery using 8/0 nylon while the cephalic vein was anastomosed with the toracodorsal vein using the same suture both in end-to-end fashion under microscope magnification (Figure 3).

A meshed split thickness skin graft was applied on the flap and a penrose drain and dressing provided. The patient had a prophylaxis antigangrene (metronidazole 400–500 mg every 48 hours) since his admission at A&E, followed by an antibiotic therapy (cephalosporin of third generation) changed according to the microbiological findings (staphylococcus aureus and enterococcus foecalis). 

Furthermore a subcutaneous injection of 40 mg of enoxaparin sodium was administrated every day for 2 weeks. 

Three days later the patient developed a recurrent infection on the dorsal distal aspect of the forearm. 

The flap was still viable and both anastomoses were patent at the hand Doppler. 

The wound was therefore explored and a further debridement was carried out to remove the residual extensor tendons and 5 cm of necrotic distal ulna, followed by a washout with the pulsed lavage system. 

**Figure 4:**
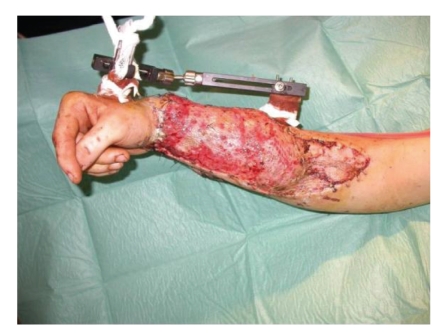
Postoperative at 2 weeks.

**Figure 5:**
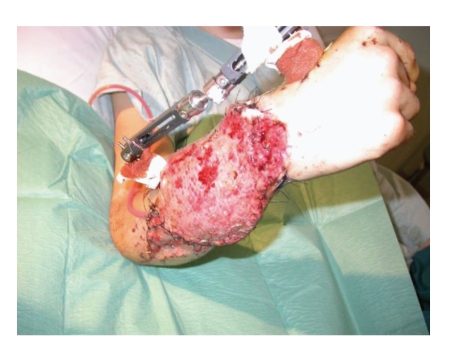
Postoperative at 2 weeks.

After surgery the patient underwent hyperbaric oxygen therapy and an aimed antibiotic therapy. The additional use of hyperbaric oxygen therapy allowed a successful healing of this complex severely contaminated wound in two weeks time (Figures 4  and 5). 

The physiotherapy treatment was immediately begun initially with static and then dynamic splints. 

**Figure 6:**
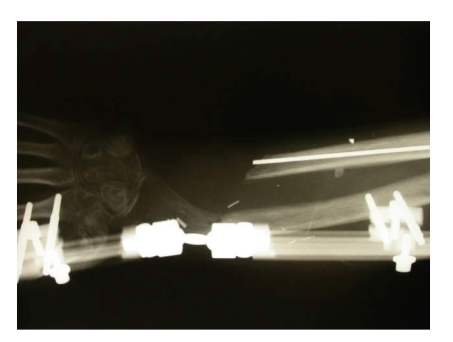
X-ray showing a further displacement of the fracture fragment of the radius, with ulnar deviation of the wrist.

The patient sustained another accident shortly after the first trauma with a complicated fracture of the radius and ulnar deviation of the wrist (Figure 6).

**Figure 7:**
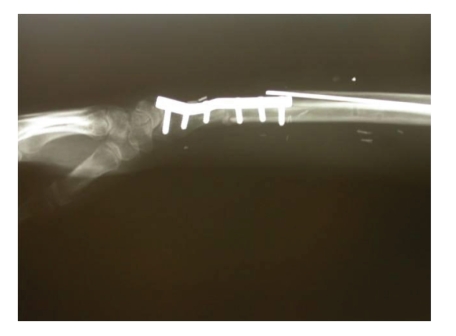
X-ray showing a reduced fracture with plates and screws.

The ortopedic surgeon removed the external fixation and reduced the fracture with plates and screws. The wrist was fixed in arthrodesis (Figure 7).

**Figure 8:**
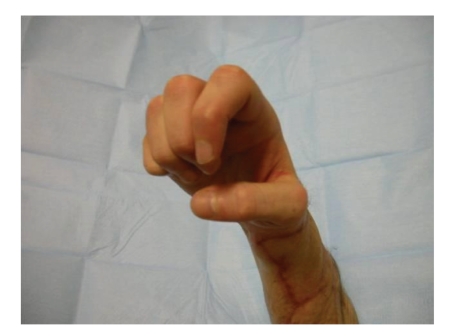
Followup at 1 year with 95% recovery of the flexor tendons.

**Figure 9:**
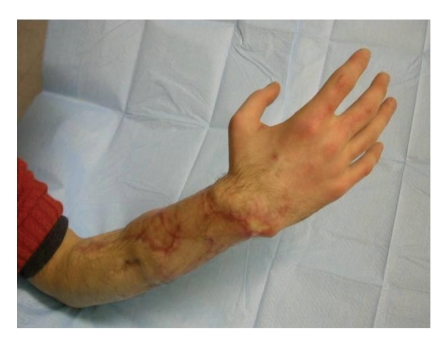
Followup at 1 year with 40% recovery of the extensor tendons.

**Figure 10:**
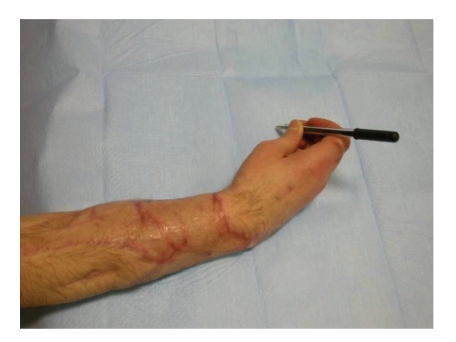
Patient able to write.

**Figure 11:**
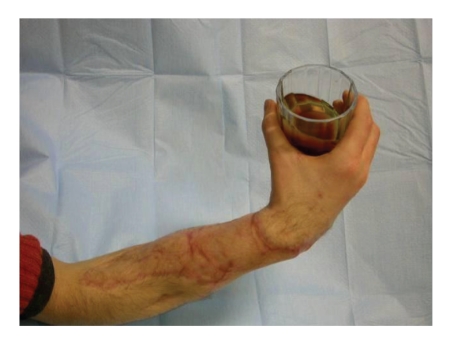
Patient able to carry a glass.

The patient underwent a further physiotherapy treatment and the functional outcome was measured in degrees of range of motion (ROM) at 1-year followup as follows: 72° of flexion at MCPJ; 70° of flexion at PIPJ; 39° of flexion at DIPJ; 1 cm away from 13° of extension at PIPJ; minus 8° of extension at DIPJ. The range of extension motion was probably due to the vicarious action of the inteosseous muscles (Figures 8, 9, 10, and 11).

A potential delayed reconstruction of the extensor tendons with a tendinous graft or transfer to increase the range of motion was discussed with the patient.

**Figure 12:**
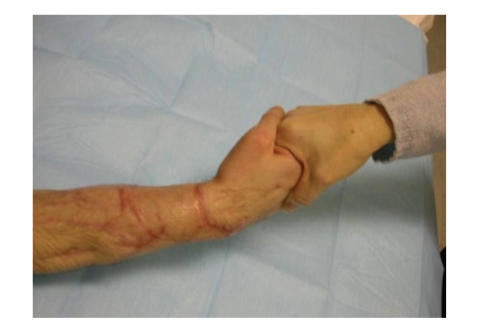
Patient showing a good strength.

However the patient was satisfied with the outcome of the operation and was able to use his hand with a reasonable strength for the daily activities, as shown in the pictures (Figure 12).

## 3. Discussion

The surgical treatment of severe infections following trauma of the upper limb frequently leaves important defects that require complex reconstructive procedures. 

Traditional concepts of wound treatment include multiple debridements followed by a long period of topical wound care, frequently supported by Vacuum-Assisted Closure [3–5], before proceeding with flap reconstruction. 

However the evolution of microsurgical techniques by using free tissue transfert has facilitated a one-stage reconstruction of these complex defects. 

Their value in infection control has been proved in several studies [6–8], which demonstrated excellent results with the early coverage of posttraumatic defects in the upper extremity. 

The advantages of free muscle flaps for reconstruction of the upper and lower extremity defects have been largely reported to be superior to those of fasciocutaneous flaps [9–11]. 

However some authors achieved equal functional outcomes with fasciocutaneous flaps [12, 13], which are usually effective for covering small medium size defects. 

Conversely free muscle or musculocutaneous flaps can provide large wound coverage for the debrided bone and soft tissue, obliterate dead spaces, improve local vascularity, and deliver oxygen to the contaminated wound. 

The authors made the LD flap the first choice in this case report due to the extensively wide soft tissue defect in which a large flap was required to cover the vital structures and the fixation device. 

The presence of a proximal interrupted radial artery with a good outflow further strengthens our choice to harvest a free flap. 

The combination of the free LD flap with hyperbaric therapy was able to successfully salvage the upper limb, especially in this complex severely contaminated wound, with a recurrent infection.

However we do not have an objective study to demonstrate in this case report the effectiveness of the hyperbaric oxygen therapy alone or combined with a specific antibiotic therapy although the information supporting the use of hyperbaric oxygen therapy for a variety of disorders, including gas gangrene, is available in literature [14]. 

Better data are therefore required to develop stronger guiding principles for individual clinical situations.

## 4. Conclusion

The benefits of the one-stage procedure, when possible, are well known. 

However it is opportune to accurately assess each case individually in order to establish a tradeoff between the necessity of an immediate coverage of the vital structures and the risk to close a potential infected wound. 

In this case report we experienced a recurrent infection and the use of a combination of a free flap with a hyperbaric oxygen therapy was able to successfully cure a severely complex contaminated wound. 

## References

[1] Guelinckx PJ, Sinsel NK (1995). Refinements in the one-stage procedure for management of chronic osteomyelitis. *Microsurgery*.

[2] Koschnick M, Bruener S, Germann G (2003). Free tissue transfer: an advanced strategy for postinfection soft-tissue defects in the upper extremity. *Annals of Plastic Surgery*.

[3] Fleischmann W, Lang E, Russ M (1997). Treatment of infection by vacuum sealing. *Unfallchirurg*.

[4] Joseph E, Hamori CA, Bergman S, Roaf E, Swann NF, Anastasi GW (2000). A prospective randomized trial of vacuum-assisted closure versus standard therapy of chronic nonhealing wounds. *Wounds*.

[5] Argenta LC, Morykwas MJ (1997). Vacuum-assisted closure: a new method for wound control and treatment: clinical experience. *Annals of Plastic Surgery*.

[6] Godina M (1986). Early microsurgical reconstruction of complex trauma of the extremities. *Plastic and Reconstructive Surgery*.

[7] Lister G, Scheker L (1988). Emergency free flaps to the upper extremity. *The Journal of Hand Surgery*.

[8] Breidenbach WC (1989). Emergency free tissue transfer for reconstruction of acute upper extremity wounds. *Clinics in Plastic Surgery*.

[9] Mathes SJ, Alpert BS, Chang N (1982). Use of the muscle flap in chronic osteomyelitis: experimental and clinical correlation. *Plastic and Reconstructive Surgery*.

[10] Eshima I, Mathes SJ, Paty P (1990). Comparison of the intracellular bacterial killing activity of leukocytes in musculocutaneous and random-pattern flaps. *Plastic and Reconstructive Surgery*.

[11] Gosain A, Chang N, Mathes S, Hunt TK, Vasconez L (1990). A study of the relationship between blood flow and bacterial inoculation in musculocutaneous and fasciocutaneous flaps. *Plastic and Reconstructive Surgery*.

[12] Yazar S, Lin C-H, Lin Y-T, Ulusal AE, Wei F-C (2006). Outcome comparison between free muscle and free fasciocutaneous flaps for reconstruction of distal third and ankle traumatic open tibial fractures. *Plastic and Reconstructive Surgery*.

[13] Chen H-C, Tang Y-B, Mardini S, Tsai B-W (2004). Reconstruction of the hand and upper limb with free flaps based on musculocutaneous perforators. *Microsurgery*.

[14] Hirn M (1993). Hyperbaric oxygen in the treatment of gas gangrene and perineal necrotizing fasciitis. A clinical and experimental study. *European Journal of Surgery, Supplement*.

